# Zoonotic *Cryptosporidium* and *Giardia* in marsupials—an update

**DOI:** 10.1007/s00436-024-08129-w

**Published:** 2024-01-23

**Authors:** Amanda D. Barbosa, Siobhon Egan, Yaoyu Feng, Lihua Xiao, Samson Balogun, Una Ryan

**Affiliations:** 1 Harry Butler Institute, Vector- and Water-Borne Pathogens Research Group, Murdoch University, Murdoch, Western Australia 6150 Australia; 2 CAPES Foundation, Ministry of Education of Brazil, Brasilia, DF 70040-020 Brazil; 3 Guangdong Laboratory for Lingnan Modern Agriculture, Center for Emerging and Zoonotic Diseases, College of Veterinary Medicine, South China Agricultural University, Guangzhou, 510642 China; 4 Institute of Biological, Environmental and Rural Sciences, Aberystwyth University, Wales, United Kingdom

**Keywords:** *Cryptosporidium*, *Giardia*, Marsupials, Zoonotic potential, Molecular typing, Epidemiology

## Abstract

Marsupials, inhabiting diverse ecosystems, including urban and peri-urban regions in Australasia and the Americas, intersect with human activities, leading to zoonotic spill-over and anthroponotic spill-back of pathogens, including *Cryptosporidium* and *Giardia.* This review assesses the current knowledge on the diversity of *Cryptosporidium* and *Giardia* species in marsupials, focusing on the potential zoonotic risks. *Cryptosporidium fayeri* and *C. macropodum* are the dominant species in marsupials, while in possums, the host-specific possum genotype dominates. Of these three species/genotypes, only *C. fayeri* has been identified in two humans and the zoonotic risk is considered low. Generally, oocyst shedding in marsupials is low, further supporting a low transmission risk. However, there is some evidence of spill-back of *C. hominis* into kangaroo populations, which requires continued monitoring. Although *C. hominis* does not appear to be established in small marsupials like possums, comprehensive screening and analysis are essential for a better understanding of the prevalence and potential establishment of zoonotic *Cryptosporidium* species in small marsupials. Both host-specific and zoonotic *Giardia* species have been identified in marsupials. The dominance of zoonotic *G. duodenalis* assemblages A and B in marsupials may result from spill-back from livestock and humans and it is not yet understood if these are transient or established infections. Future studies using multilocus typing tools and whole-genome sequencing are required for a better understanding of the zoonotic risk from *Giardia* infections in marsupials. Moreover, much more extensive screening of a wider range of marsupial species, particularly in peri-urban areas, is required to provide a clearer understanding of the zoonotic risk of *Cryptosporidium* and *Giardia* in marsupials.

## Introduction

Marsupials (Metatherians) are non-placental mammals that diverged from placental mammals ~ 160 million years ago, which give birth to relatively undeveloped offspring and carry them in a pouch (Luo et al. 2011; Bi et al. 2018). Previously, marsupial development was considered “primitive” and perhaps ancestral. However, this is currently under debate (Wilson 2023). Modern marsupials inhabit a wide range of ecosystems but are confined to Australasia (primarily Australia and New Guinea) and the Americas (South America and secondarily North America) (Eldridge et al. 2019). The diversity of marsupials is much greater in Australasia with > 248 species (4 orders and 18 families), than in the Americas, with ~ 111 species (3 orders and 3 families). Only the Virginia opossum (*Didelphis virginiana*) is found in North America (Wilson et al. 2021). Marsupial populations in Australasia and the Americas have been separated for ~ 50–69 million years (Nilsson et al. 2004; Long 2017).

Marsupials make substantial contributions to ecosystem processes and overall health, including soil fertilization, pollination, and seed dispersal, promoting plant diversity and regeneration and spread of plant species in disturbed or degraded landscapes. Additionally, digging marsupials increase soil aeration and nutrient cycling, reducing leaf litter cover and depth, and even potentially reducing fuel loads and fire risk. Furthermore, some marsupials consume insects and other invertebrates, acting as natural pest controllers and aiding in crop protection (Isaac et al. 2014; Ryan et al. 2020; de Camargo et al. 2022). However, many marsupial populations are in decline, particularly Australasia, with 17 extinctions, 30% (74/248) of species listed as “threatened” and 14% (35/248) listed as “near threatened” by the International Union for the Conservation of Nature (IUCN) (Woinarski and Fisher 2023).

Conservation strategies including wildlife corridors, translocations and “rewilding” (Sweeney et al. 2019) require a thorough understanding of the health of marsupials and the disease risk, parasite load, and zoonotic potential of pathogens in these animals (Dunlop and Watson 2022). In addition, marsupial movements into urban and peri-urban areas to take advantage of increased food and water resources (Mackenstedt et al. 2015; Hillman and Thompson 2016) may also result in higher population densities than their normal environment, which in turn is associated with nutritional stress and higher parasite prevalence (Brandimarti et al. 2021).

The protozoan parasites *Cryptosporidium* and *Giardia* have a wide host range including marsupials and humans and can cause diarrhoeal illlness, weight loss, and malnutrition (Ryan et al. 2021). Transmission is via *Cryptosporidium* oocysts and *Giardia* cysts shed in faeces, which can persist and remain viable in the environment for months. Overlapping of marsupial habitats with human activities can result in both spill-over of zoonotic pathogens from animals to humans or other potential reservoir hosts and anthroponotic spill-back of ‘human’ parasites to naïve animal hosts (Ellwanger and Chies 2021). Oocysts and cysts shed by these animals may contaiminate drinking source water, posing potential health threat to humans. This review examines current knowledge on the diversity of *Cryptosporidium* and *Giardia* species in marsupials, with a focus on zoonotic risk and evidence for spill-over and spill-back of these parasites between marsupials and humans.

## *Cryptosporidium* spp. in marsupials

Over 169 *Cryptosporidium* species and genotypes have been described, with *C. hominis* and *C. parvum* responsible for the majority of human infections (Prediger et al. 2021; Ryan et al. 2021; Yang et al. 2021; Chen et al. 2023; Garcia-R and Hayman 2023; Huang et al. 2023; Tůmová et al. 2023). The majority (~ 200) of the > 280 species of marsupials are in Australia/New Guinea, with the remainder inhabiting South America (and secondarily North America), and the two populations have been separated for ~ 50–69 million years (Nilsson et al. 2004; Long 2017). In Australia, marsupials are the dominant species inhabing catchments (Power 2010; Ryan and Power 2012) and the two main *Cryptosporidium* species infecting Australian marsupials are *C. fayeri* (previously marsupial genotype 1/koala genotype) (Ryan et al. 2008) and *C. macropodum* (previously marsupial genotype II/EGK2/EGK3) (Power and Ryan 2008). Very little is known about *Cryptosporidium* species in South/North American marsupials, but opossum genotypes 1 and 2 have been identified in opossums (*Didelphis virginiana*) (Xiao et al., 2002; Feng et al. 2007) and *C. fayeri* was putatively identified in an opossum by RFLP in another study but not confirmed by sequencing (Ziegler et al. 2007). Opossum genotype 1 is genetically very closely related to *C. fayeri* (only 0.38% divergent at the 18S rRNA locus) and is now considered a subtype of *C. fayeri* (Xiao et al. 2002; Ryan et al. 2008; Feng et al. 2007), suggesting the species was present in marsupials prior to continental drift (Ryan et al. 2008). *Cryptosporidium fayeri* has also been reported in Linnaeus’s mouse opossum in Brazil (Fehlberg et al. 2021). There have been no reports of *C. macropodum* in South/North American marsupials.

*Cryptosporidium fayeri* is largely confined to marsupial hosts but there have been two reports in humans (Waldron et al. 2010; Braima et al. 2021). *Cryptosporidium fayeri* was identified in an immunocompetent woman of 29 years in age with prolonged gastroenteritis in New South Wales (NSW) (Waldron et al. 2010), and more recently in a immunosuppressed female aged 39 years with diarrhea and acute myeloid leukaemia in Western Australia (WA) (Braima et al. 2019). Although *C. fayeri* is considered a very minor human pathogen, it has been included in Table 1, which details all reports of human infectious *Cryptosporidium* and *Giardia* in marsupials.

**Table 1:** Zoonotic *Cryptosporidium* and *Giardia* species and assemblages reported in marsupials globally

Species/assemblage	Hosts	Reference	Prevalence	Country
*Cryptosporidium*				
*C. hominis*				
	Common brushtail possums (*Trichosurus vulpecula*)	Hill et al. 2008	2.3% (5/221)	Australia
	Eastern gray kangaroos (*Macropus giganteus*)	Ng et al. 2011	11.2% (18/160)	Australia
	Southern brown bandicoot (*Isoodon obesulus*)	Dowle et al. 2013	1% (1/104)	Australia
	Swamp wallaby* (Wallabia bicolor)*	Koehler et al. 2016b	0.5% (2/388)	Australia
	Eastern gray kangaroos (*Macropus giganteus*)	Zahedi et al. 2018	3.1% (26/835)	Australia
*C. parvum*				
	Eastern gray kangaroos (*Macropus giganteus*)	Ng et al. 2011	4.4% (7/160)	Australia
	Wallaby (species unknown)	Ng et al. 2011	5.8% (1/17)	Australia
	Southern brown bandicoot (*Isoodon obesulus*)	Dowle et al. 2013	2.9% (3/104)	Australia
	Eastern gray kangaroos (*Macropus giganteus*)	Nolan et al. 2013	0.2% (1/485)	Australia
	Eastern gray kangaroos (*Macropus giganteus*)	Koehler et al. 2016b	0.07% (1/1455)	Australia
	Linnaeus’s mouse opossum (*Marmosa murina*)	Fehlberg et al. 2021	7.7% (2/26)	Brazil
*C. fayeri*				
	Koala (*Phascolarctos cinereus*)	Morgan et al. 1997; 1999	100% (1/1)	Australia
	Red kangaroo (*Macropus rufus)*	Morgan et al. 1997	100% (1/1)	Australia
	Opossum (*Didelphis virginiana*)	Xiao et al. 2002	100% (2/2)	US
	Eastern gray kangaroos (*Macropus giganteus*)	Power et al. 2005	4.1% (146/3557)	Australia
	Western gray kangaroos (*Macropus fuliginosus*)	McCarthy et al. 2008	2.7% (2/72)	Australia
	Yellow-footed rock wallaby (*Petrogale xanthopus*),	Power et al. 2009	100% (1/1)	Australia
	Western barred bandicoot (*Perameles bougainville*)	Power and Ryan 2008	100% (1/1)	Australia
	Western gray kangaroos (*Macropus fuliginosus*)	Yang et al. 2011	0.9% (7/763)	Australia
	Eastern gray kangaroos (*Macropus giganteus*)	Nolan et al. 2013	0.4% (2/485)	Australia
	Eastern gray kangaroos (*Macropus giganteus*)	Koehler et al. 2016b	0.1% (2/1455)	Australia
	Common wombat (*Vombatus ursinus*)	Koehler et al. 2016b	1.6% (7/435)	Australia
	Tasmanian devil (*Sarcophilus harrisii*)	Wait et al. 2017	0.9% (2/216)*	Australia
	Eastern gray kangaroos (*Macropus giganteus*)	Zahedi et al. 2018	0.2% (2/835)	Australia
	Western gray kangaroos (*Macropus fuliginosus*)	Zahedi et al. 2018	6.5% (156/2393)	Australia
	Sugar glider (*Petaurus breviceps*)	Takaki et al. 2020	50% (1/2)	Japan
	Linnaeus’s mouse opossum (*Marmosa murina*)	Fehlberg et al. 2021	3.8% (1/26)	Brazil
*C. cuniculus*				
	Eastern gray kangaroo (*Macropus giganteus*)	Koehler et al. 2014	100% (1/1)	Australia
	Eastern gray kangaroos (*Macropus giganteus*)	Koehler et al. 2016b	0.1% (2/1455)	Australia
*C. meleagridis*				
	Brushtailed rock wallaby (*Petrogale pencillata*)	Vermeulen et al. 2015a	1.5% (5/324)	Australia
*C. muris*				
	Bilbies (*Macrotis lagotis*)	Warren et al. 2003	39.3% (11/28)	Australia
	Tasmanian devil (*Sarcophilus harrisii*).	Wait et al. 2017	0.5% (1/216)	Australia
*C. ubiquitum*				
	Brushtailed rock wallaby (*Petrogale pencillata*)	Vermeulen et al. 2015a	0.6% (2/324)	Australia
	Common wombat (*Vombatus ursinus*)	Koehler et al. 2016a; 2016b	0.5% (2/435)	Australia
*C. xiaoi*				
	Western gray kangaroos (*Macropus fuliginosus*)	Yang et al. 2011	0.8% (6/763)	Australia
	Eastern gray kangaroos (*Macropus giganteus*)	Nolan et al. 2013	0.4% (2/485)	Australia
*Giardia*				
Assemblage A				
	Western gray kangaroos (*Macropus fuliginous*)	McCarthy et al. 2008	4.2% (3/72)	Australia
	Common brushtail possum (*Trichosurus vulpecula)*	Thompson et al. 2008	22.9% (11/48)	Australia
	Mountain brushtail possum (*Trichosurus cunninghami*)	Thompson et al. 2008	21.9% (7/32)	Australia
	Rufous bettong (*Aepyprymnus rufescens*)	Thompson et al. 2008	50% (1/2)	Australia
	Western gray kangaroos (*Macropus fuliginous*)	Thompson et al. 2008	2.9% (4/136)	Australia
	Southern hairy-nosed wombat (*Lasiorhinus latifron*s)	Thompson et al. 2008	20% (1/5)	Australia
	Long-nosed potoroo (*Potorous tridactylus*)	Thompson et al. 2008	33.3% (1/3)	Australia
	Red kangaroo (*Macropus rufus)*	Thompson et al. 2008	1.8% (1/55)	Australia
	Parma wallaby (*Macropus parma*)	Thompson et al. 2008	20% (1/5)	Australia
	Tammar wallaby (*Macropus eugeni*)	Thompson et al. 2008	4.2% (1/24)	Australia
	Yellow-footed rock-wallaby (*Petrogale xanthopus*)	Thompson et al. 2008	100% (1/1)	Australia
	Koala (*Phascolarctos cinereus*)	Thompson et al. 2008	2.5% (1/40)	Australia
	Southern brown Bandicoot (Quenda) Southern brown Bandicoot (Quenda) (*Isoodon obesulus*)	Thompson et al. 2010	3.6% (2/55)	Australia
	Common planigale (*Planigale maculata*)	Thompson et al. 2010	20% (1/5)	Australia
	Swamp wallaby (*Wallabia bicolor*)	Thompson et al. 2010	5% (1/20)	Australia
	Eastern gray kangaroos (*Macropus giganteus*)	Ng et al. 2011	5% (8/160)	Australia
	Common wombat (*Vombatus ursinus*)	Nolan et al. 2013	3.3% (6/181)	Australia
	Brushtailed rock wallaby (*Petrogale pencillata*)	Vermeulen et al. 2015b	3.5% (11/318)	Australia
	Eastern gray kangaroos (*Macropus giganteus*)	Koehler et al. 2016b	0.07% (1/1455)	Australia
	Common wombat (*Vombatus ursinus*)	Koehler et al. 2016b	0.3% (1/435)	Australia
	Southern brown Bandicoot (Quenda) (*Isoodon obesulus*)	Hillman et al. 2019	1.6% (1/63)*	Australia
	Eastern gray kangaroos (*Macropus giganteus*)	Zahedi et al. 2020	3.9% (33/838)	Australia
	Northern three-striped opossum (*Monodelphis americana*)	Fehlberg et al. 2021	12.5% (1/8)	Brazil
	Agile gracile opossum (*Gracilinanus agilis*),	Fehlberg et al. 2021	12.5% (1/8)	Brazil
Assemblage B				
	Eastern gray kangaroos (*Macropus giganteus*)	Ng et al. 2011	1.2% (2/160)	Australia
	Koala (*Phascolarctos cinereus*)	Thompson et al. 2008	7.5% (3/40)	Australia
	Spotted quoll (*Dasyurus maculatus*	Thompson et al. 2008	33.3% (1/3)	Australia
	Western gray kangaroos (*Macropus fuliginosus*)	Thompson et al. 2008	2.2% (3/136)	Australia
	Parma wallaby (*Macropus parma*)	Thompson et al. 2008	20% (1/5)	Australia
	Tammar wallaby (*Macropus eugeni*)	Thompson et al. 2008	8.3% (2/24)	Australia
	Red kangaroo (*Macropus rufus)*	Thompson et al. 2008	1.8% (1/55)	Australia
	Brushtailed rock wallaby (*Petrogale pencillata*)	Vermeulen et al. 2015b	1.6% (5/318)	Australia
	Tasmanian devil (*Sarcophilus harrisii*)	Wait et al. 2017	0.5% (1/191)	Australia
	Eastern gray kangaroos (*Macropus giganteus*)	Zahedi et al. 2020	2.5% (13/838)	Australia
A + B mixtures				
	Quokka (*Setonix brachyurus*)	Thompson et al. 2008	6.6% (1/15)	Australia
	Red kangaroo (*Macropus rufus)*	Thompson et al. 2008	5.5% (3/55)	Australia
	Swamp wallaby (*Wallabia bicolor*)	Thompson et al. 2010	5% (1/20)	Australia

^*^Co-infection with Tasmanian devil genotype

*Cryptosporidium macropodum* has been reported in eastern gray kangaroos (Power et al. 2004; Ng et al. 2012; Nolan et al. 2013; Zahedi et al. 2016, 2018), red kangaroos (Ryan and Power 2012), swamp wallabies (Ryan and Power 2012; Koehler et al. 2016b), and an albino kangaroo in a zoo in China (Zhang et al. 2021). The prevalence of *C. macropodum* is variable but has been reported at ~1% (14/1455) (Koehler et al. 2016a, b), 1.4% (7/485) (Nolan et al. 2013), ~ 2.6% (93/3557) (Power et al. 2005) and 5.3% (44/835) (Zahedi et al. 2016, 2018) of eastern gray kangaroos, 30% (3/10) in captive red kangaroos and 60% (6/10) of captive western gray kangaroos (Thompson 2007), and 1.0% (4/388) in wallabies (Koehler et al. 2016a, b). *Cryptosporidium macropodum* was the dominant species in eastern gray kangaroos in two studies in Melbourne, Victoria (Nolan et al. 2013; Koehler et al. 2016b) (58.3% and 66.6% of positives respectively), and was also the dominant species in swamp wallabies (83% of positives, 5/6), although wombats were mainly infected with *C. fayeri* (77.7% of positives, 7/9) (Koehler et al. 2016b)*. Cryptosporidium macropodum* was also the dominant species in eastern gray kangaroos in Sydney (95.6% of positives - 44/46) (Zahedi et al. 2018), although an earlier study reported *C. fayeri* as the dominant species (61.1% of positives—146/239) compared to *C. macropodum* (38.9% of positives, 93/239) (Power et al. 2005). In western gray kangaroos, *C. macropodum* was also the dominant species (58.1% of positives, 216/372) (Zahedi et al. 2018). As with *C. fayeri*, infections with *C. macropodum* were asymptomatic, even when oocyst shedding was high (Power et al. 2005). There have been no reports of *C. macropodum* in humans.

In addition to *C. fayeri* and *C. macropodum*, the host-adapted brushtail possum genotype (Hill et al. 2008), kangaroo genotype I (Yang et al. 2011; Koehler et al. 2016b) and Tasmanian devil genotype I (Wait et al. 2017), as well as *C. galli* (Wait et al. 2017) have been identified in marsupials. In common brushtail possums, *Cryptosporidium* prevalence ranged from 5.6% (1/18) in non-urban populations to 11.3% (15/133) for urban brushtail possums (Hill et al. 2008). Sequencing was successful in 13 of the positives and a distinct host-adapted genotype, the brushtail possum genotype, was identified in 3.6% (8/221) of samples (61.5% of sequenced positives) (Hill et al. 2008), with *C. hominis*-like sequences detected in the remaining five positives successfully sequenced (Hill et al. 2008). The brushtail possum genotype has also been detected in 1.7% (1/58) of brushtail possums in the Northern Territory (NT) (Barbosa et al. 2017).

In Tasmanian devils, the overall prevalence of *Cryptosporidium* in wild and captive (intensive captive and free-range captive) populations was 19.0% (41/216), with a significant difference in prevalence among the wild population (45.1%), intensive captive (8.6%), and free-range captive populations (12.5%) (Wait et al. 2017). A novel *Cryptosporidium* genotype (Tasmanian devil genotype 1) was detected in 90.2% (37/41) of positive samples, along with a mixed Tasmanian devil genotype 1 and *C. fayeri* in one sample (1/41), and individual samples with *C. fayeri* (2.4%—1/41), *C. muris* (2.4%—1/41), and *C. galli* (2.4%—1/41) (Wait et al. 2017).

## Zoonotic *Cryptosporidium* species in marsupials

The zoonotic *Cryptosporidium* species detected in marsupials include *C. hominis*, *C. parvum*, *C. meleagridis*, *C. cuniculus*, *C. ubiquitum*, *C. muris*, and potentially zoonotic *C. xiaoi* and *C. fayeri* in order of zoonotic importance (Table 1). Several studies have detected *C. parvum* and *C. hominis* in marsupials (Table 1). For example, in possums in Sydney, *C. hominis/C. parvum*-like sequences were detected in 5 samples (Hill et al. 2008). In the Sydney catchment area, *C. hominis* and *C. parvum* were detected in eastern gray kangaroos at a prevalence of 12.2% and 4.4% respectively, representing 66% (18/27) and 25.9% (7/27) of all positives (Ng et al 2011). Additionally, *C. parvum* was detected in a single wallaby (Ng et al. 2011). In another study in NSW, *C. hominis*-like (*n* = 1) and *C. parvum*-like (*n* = 3) sequences were detected in free-ranging and captive brown bandicoots and long-nosed bandicoots (*Perameles nasuta*) (Dowle et al. 2013). In the latter three studies, however, amplification at other loci, including the glycoprotein 60 (*gp60*) and actin genes, was not successful due to low numbers of oocysts in the samples (Hill et al. 2008; Ng et al. 2011; Dowle et al. 2013), suggesting very light infections and/or mechanical carriage. In the study by Hill et al. (2008), possums in which *C. hominis/C. parvum*-like sequences were detected were shedding 0-100 oocysts per gram of faeces (OPG) (Hill et al. 2008). *Cryptosporidium parvum* was also detected in eastern gray kangaroos (Nolan et al. 2013; Koehler et al. 2016b) and two opossums in Brazil (Fehlberg et al. 2021).

More recently, *C. hominis* was detected in one swamp wallaby in Victoria and was typed as *C. hominis* subtype IbA10G2 (Koehler et al. 2016b). In another study, *C. hominis* was detected in 3.1% (26/835) of eastern gray kangaroos in Sydney catchments and was also typed as *C. hominis* IbA10G2 (Zahedi et al. 2016; 2018). In the study by Zahedi et al. (2016), the kangaroos shedding *C. hominis* oocysts were shedding median OPG of 6,514 (range 131–16,890 OPG), suggesting an actual infection as opposed to a mechanical infection (Zahedi et al. 2016; Zahedi et al. 2018). In addition, next-generation amplicon sequencing analysis of *C. hominis* infections in kangaroos at the hypervariable *gp60* locus, identified a single subtype (compared to high diversity of *C. parvum* subtypes in cattle), potentially indicating a “single recent introduction of *C. hominis* into kangaroos” (Zahedi et al. 2017).

Higher rates of *C. hominis* oocyst shedding in kangaroos suggests that *C. hominis* infections may have become established in kangaroo populations via spill-back from human activities, which agrees with the expansion of *C. hominis* into other non-human hosts in some areas (Widmer et al. 2020). For example, *C. hominis* is the dominant species infecting donkeys, is common in horses, and recognized as endemic in equids (Li et al. 2019; Wang et al. 2020; Widmer et al. 2020). The *C. hominis* in donkeys belongs to the *C. hominis* Ik subtype family, which was though to be specific to equids, but has been reported in a 9-year old girl and a 38-year old woman, both from Sweden and both experiencing diarrhea (Lebbad et al. 2018). In that study, identical *C. hominis* Ik hypervariable glycoprotein 60 (*gp60*) sequences were identified in both patients but had two fewer TCA repeats (new sutype IkA18G1), compared to the commonly reported IkA20G1 subtype in equids (Lebbad et al. 2018). The *C. hominis* IbA10G2 subtype detected in a wallaby in Victoria (Koehler et al. 2016b) and kangaroos in Sydney catchments (Zahedi et al. 2016; 2018) is a major outbreak subtype responsible for the majority of waterborne outbreaks typed to date (Zahedi and Ryan 2020; Yang et al. 2021). It has recently been proposed that *C. hominis* subtype IbA10G2 be assigned the sub-species name *C. hominis aquapotentis*, as it is a major cause of waterborne outbreaks in high-income countries with “long-standing high sanitation and water quality indices” (Tichkule et al. 2022).

*Cryptosporidium cuniculus* has been reported in eastern gray kangaroos in Victoria (subtype VbA26) (Koehler et al. 2014; 2016) and *C. meleagridis* was identified in five rock wallabies (*gp60* subtype families IIIb and IIIg) in NSW (Vermeulen et al. 2015a). *Cryptosporidium muris* has been detected in 0.5% of Tasmanian devils and in bilbies (39.3%—11/28) in a captive breeding colony (Warren et al. 2003). *Cryptosporidium ubiquitum* was identified in two brushtailed rock wallabies in Sydney (Vermeulen et al. 2015a), and a novel *C. ubiquitum* subtype (XIIg) was identified in two common wombats in Victorian catchments (Koehler et al. 2016a; 2016b). *Cryptosporidium xiaoi* has been reported in both western and eastern gray kangaroos (Yang et al. 2011; Nolan et al. 2013). These reports were all based on molecular characterization and only the study by Warren et al. (2003) confirmed the presence of oocysts in faeces by microscopy, where the high prevalence of *C. muris* in bilbies (39.3%) was thought to be due to rodent transmission combined with high stocking densities (Warren et al. 2003).

In Australia, *C. fayeri* has been detected in many marsupials, including eastern and western gray kangaroos, red kangaroos, koalas, wombats, wallabies, bandicoots, Tasmanian devils, a sugar glider and opossums (Table 1). The prevalence of verified *C. fayeri* infections in marsupials is low (0.1–6.5%) (Table 1). *Cryptosporidium fayeri* infection is generally asymptomatic in adult kangaroos (Power et al. 2005), but in one report of *C. fayeri* in a 3-month-old sugar glider in Japan, the animal presented with diarrhea and weight loss and was negative for *Clostridium perfringens*, *Salmonella*, and *Giardia,* suggesting the diarrhoea may have been due to *C. fayeri* (Takaki et al. 2020).

Subtyping of *C. fayeri* using the *gp60* locus has identified eight *gp60* subtype families (IVa-IVh) (Power et al. 2009; Takaki et al. 2020), including a novel *C. fayeri* subtype family (IVh) (subtype IVhA13G2T1) identified in a sugar glider from a Japanese pet shop (Takaki et al. 2020). In the two human cases with *C. fayeri* infections, subtypes IVaA9G4T1R1 and IVgA10G1T1R1 were detected in the NSW and WA cases respectively (Waldron et al. 2010; Braima et al. 2021). Subtype IVaA9G4T1R1 has previously been reported in eastern gray kangaroos inhabiting the main drinking water catchment for Sydney (Power et al. 2009) and subtype IVgA10G1T1R1 has been reported in kangaroos and rabbits in drinking water catchments in WA and NSW (Zahedi et al. 2016, 2018).

## *Cryptosporidium* oocyst shedding in marsupials

Oocyst shedding in marsupials is variable with oocyst shedding in kangaroos generally low but still higher than in small marsupials. One study in Sydney catchments reported an OPG/faeces range of 10 to > 50,000 OPG in kangaroos compared to a range of 10–2010 OPG in possums and 40–4900 OPG in a yellow footed rock wallaby (Power et al. 2003). Another study in Sydney catchments reported very low shedding in kangaroos (0–1257 OPG) and a median of 54 OPG in possums (range 20–89 OPG) (Cox et al. 2005). In brushtail possums, urban possums shed higher numbers of oocysts (mean of 70,581 OPG), but most were shedding low numbers (median of 25 OPG), compared to 10 OPG in a single non-urban possum screened (Hill et al. 2008). In bandicoots, mean OPG/faeces was very low ~ 43 OPG (range 0–100) (Dowle et al. 2013). A study on oocyst shedding in eastern gray kangaroos using immunomagnetic separation (IMS) combined with flow cytometry reported that oocyst shedding in most kangaroos was also low (10–50 OPG/faeces), with a small number of samples containg high numbers, with a mean of 59,948 OPG/faeces (range 20—2.0 × 10^6^ OPG) (Power et al. 2005). A later study of eastern gray kangaroos using qPCR reported an overall median OPG of 16,018 (range, 11–275,042 OPG) (Zahedi et al. 2018).

## *Giardia* spp. in marsupials

The prevalence of *Giardia* species in marsupials is variable with prevalence ranging from 0.07 (Koehler et al. 2016b) to 67.3% (Hillman et al. 2016). A study of *Giardia* in captive and wild marsupials from Western Australia, Victoria, and South Australia reported no difference in prevalence between captive (13.4%—28/209) and wild marsupials (13.7%—29/212) (Thompson et al. 2008). Similarly, in brushtailed rock wallabies from New South Wales, an overall prevalence of 22% was recorded (70/318), with no difference in prevalence between wild, supplemented, and captive bred rock wallabies (Vermeulen et al. 2015b).

Currently recognized non human infectious *Giardia* species include *G. agilis* in amphibians, *G. ardeae* and *G. psittaci* in birds, *G. varani* in lizards, *G. microti* and *G. muris* in rodents, *G. cricetidarum* in hamsters, and *G. peramelis* in marsupials (Ryan et al. 2021). The species complex *G. duodenalis*, which consists of eight assemblages, infects mammals, with assemblages A and B in humans, C and D in dogs, E in ungulates, F from cats, G from rodents and assemblage H from seals (Cacciò et al. 2018; Ryan et al. 2021).

Both host-adapted and zoonotic *Giardia* species and genotypes have been identified in marsupials. *Giardia peramelis* is the dominant non-zoonotic species infecting quenda (*Isoodon fusciventer*, previously a subspecies of southern brown bandicoot *Isoodon obesulus fusciventer*), at prevalences ranging from 1.4 to 67.3% (Adams et al. 2004; Thompson et al. 2010; Hillman et al. 2016; Hillman et al. 2019), and has also been reported in 1.6% (1/62) of northern brown bandicoots, 3.4% (2/58) of common brushtail possums, 2.7% (1/37) of brushtail rabbit-rats in the Northern Territory but not in northern quolls (0/10) (Barbosa et al. 2017). *Giardia peramelis* cysts are morphologically indistinguishable from human infectious *Giardia* species/assemblages (Hillman et al. 2016). Infections with *G. peramelis* appear to be largely asymptomatic in quenda and have never been reported in humans (Hillman et al. 2019). *Giardia* assemblages A and E (Thompson et al. 2010) and a mixed assemblage of A and D have also been reported in quenda (Hillman et al. 2019) (Table 1).

In Tasmanian devils, the overall prevalence of *Giardia* in wild and captive populations was 4.2% (8/191), with a significantly higher prevalence in wild populations (24.1%), compared to free-range captive populations (1.6%) (Wait et al. 2017). Two novel *Giardia* genotypes were detected, including Tasmanian devil genotype 1 in 50% of positives (4/8) and Tasmanian devil genotype 2 in 37.5% (3/8), along with assemblage B in 1 sample (Wait et al. 2017). The Tasmanian devil genotypes have not been reported in humans and are not considered zoonotic (Wait et al. 2017).

## Zoonotic *G. duodenalis* assemblages in marsupials

The zoonotic *G. duodenalis* assemblages, A and B, have been identified in marsupials, as well as assemblages C, D, and E. There have been a few reports of *G. duodenalis* assemblages C, D, and E in humans, especially E. However, assemblages A and B are responsible for ~ 95% of human infections (Cai et al. 2021; Krumrie et al. 2022). Assemblages C, D, and E are considered largely host-specific and have not been included in Table 1. Assemblage A is the dominant assemblage in marsupials and has been detected in bandicoots, a bettong, eastern and western gray kangaroos, a koala, possums, planigale, a potoroo, opossums, red kangaroos, wallabies, and wombats (Table 1). The highest prevalence of assemblage A has been reported in long-nosed potoroo (33.3%) and possums (21.9–22.9%) (Thompson et al. 2008) (Table 1). However, relatively small numbers of animals were sampled in that study. Kangaroos and wombats have been the most extensively sampled species, with prevalences of 0.7–4.2% and 0.3–20% reported, respectively (Table 1). In one study in Brazil, assemblage A was the only *Giardia* detected in a northern three-striped opossum and an Agile gracile opossum (Fehlberg et al. 2021).

*Giardia duodenalis* assemblage B is the second most common assemblage in marsupials and has been reported in eastern gray kangaroos, koalas, a quoll, bandicoots and a single Tasmanian Devil, with mixtures of assemblages A and B reported in a quokka, red kangaroos, and a wallaby (Table 1). As with many previous studies, there was at times significant non-concordance between loci (Thompson et al. 2008; Vermeulen et al. 2015b). For example, in the study by Thompson et al. (2008), seven samples were typed as assemblage A at the 18S rRNA locus but assemblage B at the glutamate dehydrogenase (*gdh)* locus B. These samples have been listed as assemblage A and B mixtures in Table 1. There has been one report of assemblage C in eastern gray kangaroos (Ng et al. 2011) and two reports of assemblage D in eastern gray kangaroos (Ng et al. 2011) and a single quenda (Hillman et al. 2019). Assemblage E has been reported in a single quenda (Thompson et al. 2010), eastern gray kangaroo (Zahedi et al. 2020), and a red-necked wallaby (Zou et al. 2022). There has also been one report of an assemblage C and D mixture in three quendas (Thompson et al. 2010). The identification of *G. duodenalis* assemblages C and D in a few marsupials may have been mechanical carriage from wild dogs, as assemblages C and D are the dominant assemblages in canids (Cai et al. 2021). Similarly, the identification of the livestock-associated assemblage E in marsupials, is also likely due to mechanical carriage from co-grazing pastures with livestock. Little is known about cyst shedding in marsupials but mean cyst shedding per gram of faeces of 7.8 × 10^3^ (median = 6 × 10^3^) (range 1 × 10^2^–2.2 × 10^4^) was reported in eastern gray kangaroos using qPCR (Zahedi et al. 2020).

## Zoonotic risk of *Cryptosporidium* and *Giardia* in marsupials

### Cryptosporidium

The dominant *Cryptosporidium* species infecting Australian marsupials are *C. fayeri* (Table 1) and *C. macropodum* (Power et al. 2004; Ryan and Power 2012; Ng et al. 2012; Nolan et al. 2013; Koehler et al. 2016b; Zahedi et al. 2016; Zahedi et al. 2017; Zahedi et al. 2018). With the exception of two reports of *C. fayeri* in humans (Waldron et al. 2010; Braima et al. 2021), these species are marsupial-specific species. The majority of these studies have been conducted in drinking water catchments at the peri-urban interface, and while *C. fayeri* and *C. macropodum* are generally the most common species in kangaroos, recent evidence suggests that spill-back of *C. hominis* may have resulted in its establishment in kangaroo populations (Koehler et al. 2016b; Zahedi et al. 2016a, 2018b). For example, the most recent study of kangaroos in a NSW catchment reported that *C. hominis* was the second most common species in kangaroos (36.1% of positives), after *C. macropodum* (61.1% of positives) and much more prevalent than *C. fayeri* (2.7% of positives), with significant rates of oocyst shedding from the *C. hominis* infected kangaroos (Zahedi et al. 2018).

Evidence to date suggests that *C. hominis* is not yet established in possums and bandicoots. Although there have been some reports of *C. hominis* in these hosts, PCR analysis of additional loci was negative, indicating light or transient mechanical carriage (Hill et al. 2008; Dowle et al. 2013). This is despite very close contact between possums and humans, as the common brushtail possum is one of the most abundant native marsupials in urban environments (Hill et al. 2008) and the identification of higher *Cryptosporidium* prevalence in urban possums compared to non-urban populations (Hill et al. 2008). Notwithstanding this, possums are still predominantly (> 60% of positives) infected with a host-specific brushtail possum genotype (Hill et al. 2008).

The identification of *C. cuniculus*, *C. xiaoi*, *C. meleagridis* and *C. galli* in a few marsupial samples, is likely due to mechanical carriage from marsupials co-grazing land with rabbit, sheep, and bird scats present. *Cryptosporidium muris* has been identified in bilbies and Tasmanian devils (Table 1). The *C. muris* infection in the bilbies most likely came from mice, as one of four mice trapped in the enclosures was positive for *C. muris* by microscopy and PCR (Warren et al. 2003). The high prevalence of *C. muris* in bilbies was due to high stocking densities, with evidence of stress, as one sire attacked an offspring (Warren et al. 2003). Once more enclosures became available, stocking densities were reduced and seven of the 11 infected bilbies cleared the *C. muris* infection. However, four bilbies remained positive for six months, suggesting actual infections. Clinical signs were not observed in any of the infected bilbies (Warren et al. 2003). *Cryptosporidium muris* has a wide host range and while there have been many reports in humans, it is most reported in humans from low to middle income countries and in travellers to those areas (Yang et al. 2021).

There have been two reports of *C. ubiquitum* in wallabies and wombats but at low prevalence (0.6–0.5%—Table 1). *Cryptosporidium ubiquitum* has a wide host range and is more commonly reported in humans from high-income countries than low-income countries (Yang et al. 2021) but has not been reported in humans in Australia to date. It is prevalent in sheep in globally (Guo et al. 2021) including Australia (Yang et al. 2014; Zahedi et al. 2018), and the identification of *C. ubiquitum* in marsupials may have been due to mechanical carriage from co-grazing pastures from sheep.

### Giardia

The dominance of the zoonotic *G. duodenalis* assemblages A and B in marsupials may be due to spill-back from humans as interactions between humans and small marsupials are common and include humans offering food (Hillman and Thompson 2016; Hillman et al. 2017a; Hillman et al. 2017b; Hillman et al. 2019). The few subtyping studies conducted have detected the largely animal-associated sub-assemblage AI in a common planigale (Thompson et al. 2010), brushtailed rock-wallabies (Vermeulen et al. 2015b), a single eastern gray kangaroo, and wombat in a Melbourne catchment (Koehler et al. 2016b) and in eastern gray kangaroos in Sydney catchments (Ng et al. 2011; Zahedi et al. 2020). It is also possible that marsupials may be acquiring assemblage A from co-grazing with other animals in catchments. For example, in the study by Zahedi et al. (2020), assemblage AI sequences determined at the *gdh* locus in kangaroos were identical to sheep-derived sub-assemblage AI isolates from the same catchment. Evidence to date suggests that *G. duodenalis* sub-assemblage AI is largely confined to animals and is rarely found in humans (Klotz et al. 2022).

There are some technical issues with the *Giardia* typing methods used. Some studies relied on typing based on sequencing a very short (292 bp) 18S rDNA amplicon, which has very few polymorphic sites (McCarthy et al. 2008) and is therefore less reliable for assigning assemblages (Cai et al. 2020). Other studies used only one locus (Nolan et al. 2013; Koehler et al. 2016b). Since discordance of assemblage assignment using different loci is commonly reported in *Giardia* typing studies across different hosts, including in some of the marsupial studies (Thompson et al. 2008; Vermeulen et al. 2015b), multilocus typing at three loci is essential (Cacciò et al. 2018; Cai et al. 2021). In addition, high levels of mixed *Giardia* infections and allelic sequence heterozygosity (ASH) further complicate *G. duodenalis* assemblage assignment (Feng and Xiao 2011; Woschke et al. 2021; Zajaczkowski et al. 2021).

More reliable multilocus assemblage-specific typing tools have been developed and applied based on comparative genomic analysis to improve subtyping of assemblage A (Ankarklev et al. 2018; Klotz et al. 2022) and B (Wielinga et al. 2015; Seabolt et al. 2021). These methods should be applied to assemblage A and B infections in marsupials as well as assemblage A and B isolates in human in Australia, to better understand the zoonotic implications of the high prevalence of assemblage A and B in marsupials. Assemblage-specific multilocus sequence typing (MLST) tools have yet to be developed for the remaining assemblages, although genomes of assemblages E, C, and D have been sequenced (Jerlström-Hultqvist et al. 2010; Kooyman et al. 2019).

Marsupial-specific *Giardia* species are also common in some marsupial hosts, irrespective of being in urban or rural environments. For example, bandicoots, like possums are also highly adapted to urban environments but are predominantly infected with the host specific *G. peramelis* (Thompson et al. 2010; Hillman et al. 2016; Hillman et al. 2019; Barbosa et al. 2017).

## Conclusions

The host-specific *C. fayeri* and *C. macropodum* are the dominant species in marsupials, while in possums, the host-specific possum genotype dominates. Of these three species/genotypes, only *C. fayeri* has been identified in two humans and thus the zoonotic risk is low. In general, oocyst shedding in marsupials and particularly in small marsupials is low, further supporting a low transmission risk. There is however evidence of spill-back of *C. hominis* into kangaroo populations and some detections of *C. parvum* in marsupials, which needs careful monitoring moving forward. At present *C. hominis* does not appear to be established in small marsupial species such as possums and bandicoots, but much more extensive screening and analysis is required to better understand the prevalence and significance of *C. hominis* and *C. parvum* in marsupials, particularly possums and other small marsupials, as the last significant study in possums was conducted in 2008 (Hill et al. 2008). The identification of other zoonotic species of *Cryptosporidium* in marsupials, with the exception of *C. muris*, is likely due to mechanical carriage, but further studies are required to confirm this.

The dominance of *G. duodenalis* assemblage A and B in marsupials may have come from spill-back from humans or in the case of assemblage A may have been due to spill-over from sheep and other livestock into kangaroos. Extensive use of newly developed multilocus typing tools for *Giardia* and whole genome sequencing on a much wider range of large and particularly small marsupial species, especially in peri-urban areas, where marsupials and humans co-exist, and a better understanding of cyst numbers shed by small marsupials, is required before a clearer understanding of the zoonotic risk of *Giardia* in marsupials can be obtained. A more thorough understanding of the prevalence of zoonotic *Cryptosporidium* and *Giardia* in marsupials is also essential baseline data for conservation efforts.

## Data availability

Not applicable.

## Declarations

**Ethical approval**

Not applicable.
